# Bacteriophage deploys a RecA-dependent nuclease to inhibit *Staphylococcus aureus* replication and promote phage propagation

**DOI:** 10.1093/nar/gkag024

**Published:** 2026-01-20

**Authors:** Qi Xu, Neng Xu, Li Tang, Xindi Huang, Wei Tang, Mengke Li, Yangbo Hu, Yong Zhang, Shiyun Chen

**Affiliations:** State Key Laboratory of Virology and Biosafety, Wuhan Institute of Virology, Chinese Academy of Sciences, Wuhan 430071, China; State Key Laboratory of Virology and Biosafety, Wuhan Institute of Virology, Chinese Academy of Sciences, Wuhan 430071, China; State Key Laboratory of Virology and Biosafety, Wuhan Institute of Virology, Chinese Academy of Sciences, Wuhan 430071, China; State Key Laboratory of Virology and Biosafety, Wuhan Institute of Virology, Chinese Academy of Sciences, Wuhan 430071, China; State Key Laboratory of Virology and Biosafety, Wuhan Institute of Virology, Chinese Academy of Sciences, Wuhan 430071, China; State Key Laboratory of Virology and Biosafety, Wuhan Institute of Virology, Chinese Academy of Sciences, Wuhan 430071, China; State Key Laboratory of Virology and Biosafety, Wuhan Institute of Virology, Chinese Academy of Sciences, Wuhan 430071, China; State Key Laboratory of Virology and Biosafety, Wuhan Institute of Virology, Chinese Academy of Sciences, Wuhan 430071, China; State Key Laboratory of Virology and Biosafety, Wuhan Institute of Virology, Chinese Academy of Sciences, Wuhan 430071, China

## Abstract

Bacteriophages have evolved diverse strategies to manipulate host processes, yet the molecular mechanisms employed by phage-encoded effector proteins remain poorly understood. Here, we identify Gp16, an early-expressed protein from *Staphylococcus aureus* phage ΦNM1, as a RecA-dependent nuclease that plays a dual role in host inhibition and phage propagation. Gp16 is rapidly expressed upon infection, and its overexpression alone is sufficient to inhibit bacterial growth where deletion of *gp16* severely impairs phage DNA replication, progeny production, and host cell lysis, underscoring its essential role in the phage life cycle. Structural modeling predicts Gp16 is a nuclease, and its overexpression induces DNA condensation *in vivo*. Biochemical and cellular analyses show that Gp16 interacts with the host RecA protein to inhibit growth, and functions as a nickase *in vitro*, requiring the catalytic cysteine C181 for DNA cleavage. RecA further enhances its cleavage activity. During phage infection, RecA activation is required for efficient phage propagation, while Gp16 concurrently suppresses host DNA replication and promotes DNA condensation, thereby facilitating phage replication. Together, these findings reveal a previously unrecognized strategy in which a phage-encoded nuclease exploits the host RecA machinery to couple host suppression with productive phage propagation.

## Graphical Abstract

**Graphical Abstract:**
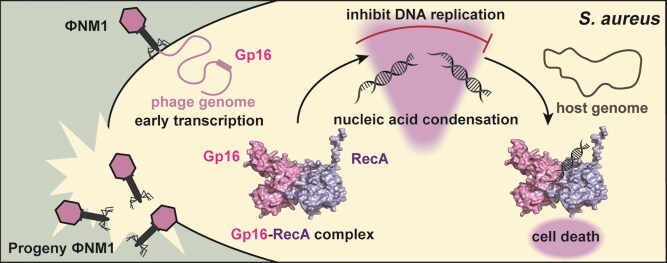

## Introduction

Bacteria and their phages engage in a continuous evolutionary arms race, in which host defense systems evolve to restrict phage infection, while phages develop countermeasures to overcome these barriers [1]. Bacteria employ various nucleic-acid-based immune systems, including restriction–modification (RM) [2], CRISPR–Cas [3], Gabija [4], and cyclic oligonucleotide-based anti-phage signaling systems (CBASS) [5], to degrade foreign DNA or trigger abortive infection responses. In response, phages have evolved diverse strategies to evade or suppress these defenses, such as genome modification, anti-CRISPR proteins [6, 7], and nucleases that directly target host defense components [8, 9]. These molecular conflicts continuously fuel the diversification of both bacterial and phage genomes, shaping their coevolutionary landscape.

Beyond mere counter-defense, bacteriophages have evolved diverse mechanisms to reprogram host physiology during infection, ensuring efficient replication within bacterial cells [10, 11]. A major target of this is reprogramming host nucleic acid metabolism. By degrading or modifying bacterial DNA and RNA, phages redirect nucleotide pools toward their own genome synthesis while suppressing host replication, transcription, and repair systems [12–14]. Such interference not only conserves energy for phage propagation but also disrupts cellular defenses, allowing the phage to dominate the host’s molecular machinery [15]. However, it remains unclear whether these observed alterations in bacterial physiology result directly from phage-encoded effectors, and how these changes ultimately favor the phage.

Phage-encoded nucleases play pivotal roles in these processes by mediating nucleic acid degradation, recombination, and genome processing [16]. Among them, HNH endonucleases are particularly widespread and functionally diverse. In many double-stranded DNA (dsDNA) phages, they act in concert with the large terminase subunit (TerL) to introduce precise nicks or double-stranded breaks during genome packaging, ensuring accurate DNA encapsulation and virion assembly [17, 18]. The HNH-type nuclease gp74 of bacteriophage HK97 exemplifies this role by promoting site-specific cleavage during head maturation [19, 20]. Beyond these packaging functions, certain HNH nucleases exploit host recombination machinery. For instance, the Ref protein of bacteriophage P1, a RecA-dependent HNH endonuclease, targets D-loop structures formed by RecA and introduces directed double-strand breaks. This represents a host-assisted mechanism of phage DNA processing, although its physiological roles are still poorly characterized [21–23]. Phage-encoded endonucleases can also participate in molecular conflicts. For example, ICP1 phages deploy a chimeric nuclease that counteracts *Vibrio cholerae* phage-inducible chromosomal islands, reflecting their adaptive role in host–phage coevolution [24]. Moreover, thermophilic phages such as *Geobacillus* virus E2 encode highly stable HNH endonucleases (GVE2 HNHE) with metal-dependent double-stranded DNA cleavage activity, emphasizing the evolutionary versatility of these enzymes across diverse ecological niches [25]. However, the substrate specificity, timing of action, and regulatory mechanisms of many nucleases remain poorly characterized.

Here, we characterize the *Staphylococcus aureus* phage protein Gp16, which promotes phage infection by interacting with the host protein RecA and concurrently inhibits host DNA replication. Our findings demonstrate that Gp16 possesses DNA cleavage activity, leading to the arrest of chromosomal replication by obstructing replication fork progression and suppressing the transcription of genes associated with replication. The elucidated mechanism offers new insights into the molecular strategies employed by phages to manipulate and exploit host cells. Our results uncover a previously unrecognized mechanism by which a phage protein modulates host DNA metabolism, shedding light on phage–host evolutionary dynamics and underscoring the potential of phage proteins as novel antibacterial targets.

## Materials and methods

### Bacterial strains and growth conditions

Bacterial strains used in this study are listed in Supplementary Table S1. *Staphylococcus aureus* was cultured in tryptic soy broth (TSB, Difco) with shaking or on tryptic soy agar (TSA, Difco) at 37°C. *Escherichia coli* strains were grown in Luria–Bertani (LB) medium and incubated at 37°C for amplifying plasmids or at 20°C for protein expression. Erythromycin (10 μg ml^−1^) or chloramphenicol (10 μg ml^−1^) was supplemented to the medium when needed for *S. aureus* and ampicillin (100 μg ml^−1^) or kanamycin (50 μg ml^−1^) for *E. coli*. To induce gene expression in *S. aureus*, 0.1 mM isopropyl β-d-1-thiogalactopyranoside (IPTG) or 10 ng ml^−1^ anhydrotetracycline (aTc) were added to the cultures.

### Plasmid constructions

All plasmids used in this study are listed in Supplementary Table S1, and all primers and synthesized gene sequences are listed in Supplementary Table S2. Genes were cloned into plasmids using either a ClonExpress II one-step cloning kit (Vazyme, China) or restriction enzymes (NEB, USA). The gene expression plasmid pTAS in *S. aureus* contains an aTc-inducible promoter. For protein expression and purification in *E. coli*, genes were cloned into pET28a or pET21 vector. Bacterial two-hybrid (BATCH) assays were performed using either a pUT18 or pKT 25 vector [26]. The gene knockout plasmid pSA-IPTG contains IPTG-inducible Cas9 and two *Bsa* I sites for sgRNA cloning. For gene complementation, the plasmid pCI was used [27].

### Mutant construction and complementation of recA in *S. aureus*

The CRISPR–Cas9 system was used to construct a *recA* deletion mutant. First, the single strand of small guide RNA (sgRNA) was synthesized, and the polymerase chain reaction (PCR) products were ligated to the pSA-IPTG vector at the *Bsa* I site by T4 DNA ligase. Next, after annealing, 1 kb upstream and downstream fragments of the *recA* ORF were amplified from *S. aureus* RN4220 genomic DNA (gDNA) and then sub-cloned into the pSA vector at the *EcoR* I and *BamH* I sites. The resulting deletion plasmid pSA-IPTG-*recA* was subsequently used to transform RN4220. Cells were plated on TSA with 1 mM IPTG, and the correct deletion colonies were screened. For gene complementation, *recA* with its 1 kb upstream fragment was cloned into the plasmid pCI and then used to transform *S. aureus*. The complementary strains were selected by plating on TSA containing 10 μg ml^−1^ chloramphenicol.

### Knockout of gp16 in the ΦNM1 phage

The *gp16*-deficient strain ΦNM1^Δgp16^ was generated by CRISPR–Cas9 system. As described above, the Cas9 and sgRNA expression was induced by IPTG from the spac-regulated promoter system. Specifically, 10 μl ΦNM1 was mixed with 100 μl RN4220 transformed with pSA-IPTG-*gp16* and incubated at room temperature for 10 min. Next, 5 ml of 0.7% top agar was added to the mixture, and then the suspensions were plated on a TSA plate containing 1mM IPTG. After incubation at 37°C for 16 h, mutant candidates were confirmed by PCR and stored in SM buffer (50 mM Tris–HCl, pH 7.8, 1 mM MgSO_4_, 4 mM CaCl_2_, 100 mM NaCl, 0.1% gelatin) at 4°C.

### Plaque and efficiency of plating assay

Plaque assays were performed by mixing 100 μl of log-phase *S. aureus* culture (OD_600_ = 0.6) with 10 μl of diluted phage suspension and 5 ml of 0.7% TSA top agar, overlaying the mixture onto TSA plates, and incubating overnight at 37°C for plaque formation. For efficiency of plating (EOP) determination, 100 μl of log-phase bacterial culture (OD_600_ = 0.6) was mixed with 5 ml of 0.7% TSA top agar and poured onto TSA plates. Two microliters of serial 10-fold phage dilutions were spotted on the double-layer agar plates and then incubated for 18 h at 37°C until plaques formed.

### Phage infection assay in liquid culture

Phage liquid infection was performed as described [28]. Overnight cultures of *S. aureus* were diluted 1:100 in fresh TSB containing a final concentration of 5 mM CaCl_2_ to OD_600_ of 0.2, and 200 μl of cells were added to each well of a 96-well plate. ΦNM1 WT or ΦNM1^Δgp16^ phages were immediately added to each well at the indicated multiplicity of infection (MOI) of 0.01, 1, and 10, respectively. After phage infection, growth was measured every 15 min at 37°C using a shaking plate reader (Biotek, USA). The growth curve experiment was replicated at least three times independently.

For quantification of plaque forming unit (PFU) and colony-forming unit (CFU), overnight cultures were diluted to an OD_600_ of 0.1 in 20 ml of TSB and incubated at 37°C with shaking (200 rpm) for 2 h. Phage infection was initiated at an MOI of 0.01 in the presence of 5 mM CaCl₂. At the indicated time points, 200 μl of the samples were collected and centrifuged at 12 000 × *g* for 2 min at 4°C. The supernatant was used for phage titration by plaque assay. The cell pellet was washed once with phosphate-buffered saline (PBS), resuspended in 150 μl of TSB, serially diluted 10-fold, and plated on TSA. Plates were incubated overnight at 37°C, and colonies were counted the following day.

### Phage lysogens generation

ΦNM1 and ΦNM1^Δgp16^ lysogens were obtained as described [29]. Briefly, phages were spotted onto 0.7% top agar overlays seeded with *S. aureus* RN4220 and incubated overnight at 37°C. Material from the center of a lysis zone was collected with a sterile inoculation loop and streaked onto TSA plates. After 12 h of incubation at 37°C, colonies were screened for prophage integration by PCR using primer pairs *attL*-F/*attL*-R and *attR*-F/*attR*-R to amplify the *attL* and *attR* junctions, respectively. A verified lysogen was retained in SM buffer (50 mM Tris–HCl, pH 7.5, 10 mM MgSO_4_, 200 mM NaCl) for subsequent experiments.

### Construction of ΦNM1-Erm or ΦNM1Δgp16-Erm

The ΦNM1-Erm^R^ and ΦNM1^Δgp16^-Erm^R^ mutants were constructed using a CRISPR–Cas9-mediated allelic exchange system. A single-stranded sgRNA was synthesized and cloned into the pSA-IPTG vector at the *BsaI* site using T4 DNA ligase. The ~1-kb homology arm flanking the ΦNM1 integration site was amplified from ΦNM1 gDNA using primer pairs *erm*-up-F/*erm*-up-R and *erm*-dn-F/*erm*-dn-R. The ~1.25 kb *ermC* resistance cassette was amplified from plasmid pTAS with primers *erm*-F/*erm*-R. The three fragments (~3.25 kb in total) were assembled by overlap PCR using external primers *erm*-up-F and *erm*-dn-R, and *attB* adapter sequences were added to enable directional cloning into pSA-IPTG. For mutant construction, 10 μl of ΦNM1 phage lysate was mixed with 100 μl of *S. aureus* RN4220 cells harboring pSA-IPTG*-erm*, and the mixture was incubated at room temperature for 10 min. The mixture was then overlaid with 5 ml of 0.7% top agar and poured onto TSA plates containing 1 mM IPTG. After incubation at 37°C for 16 h, the resulting plaques were screened for *ermC* insertion by PCR using primers *erm*-ve-F and *erm*-ve-R. Confirmed mutant phages were propagated and stored in SM buffer (50 mM Tris–HCl, pH 7.8, 1 mM MgSO_4_, 4 mM CaCl_2_, 100 mM NaCl, 0.1% gelatin) at 4°C.

### Quantification of erythromycin-resistant lysogens

As previously described [30], overnight cultures of *S. aureus* RN4220 were started from single colonies in TSB and subsequently diluted 1:100 into fresh TSB supplemented with 5 mM CaCl_2_ before incubation at 37°C with shaking for 1 h. Cells were then exposed to either ΦNM1-Erm or ΦNM1^Δgp16^-Erm at an MOI of ~10 and incubated on ice for 30 min to facilitate adsorption. Next, cultures were transferred to 37°C and incubated with aeration for 4 h. Serial dilutions were subsequently plated onto TSA plates containing 5 mM CaCl_2_ with or without erythromycin, and lysogen formation was quantified by colony counts.

### Predicted protein structure

The primary sequences of Gp16 and RecA were submitted to AlphaFold3 (AF3) for structure prediction [31]. To model the Gp16–RecA complex, the sequences of both proteins were provided as input to AF3 using the multimer mode to predict the protein–protein complex. Structural comparisons and visualizations were performed in PyMOL (https://pymol.org/).

### Microscopy and image analysis

Overnight cultures of *S. aureus* cells were diluted 1:100 into TSB containing 10 ng ml^−1^ aTc and grown at 37°C by shaking at 200 rpm. Cells were harvested every 2 h by centrifugation at 5000 rpm for 3 min, and then washed twice and resuspended in 10 μl PBS. Cells were mounted on glass slides and observed under an Olympus inverted microscope with a 100× oil immersion phase contrast objective. Images were analyzed using ImageJ [32].

### Nucleoid staining

The nucleoid staining was performed as described [33]. In brief, cells were stained with 10 μM 4′,6-diamidino-2-phenylindole (DAPI) for 10 min at room temperature. Next, 2 μl of the mixture was flipped on to a microscope glass slide for imaging. The labeled cells were observed using phase contrast and the DAPI channel on an Olympus microscope.

### Protein pull-down in *S. aureus* and LC–MS

The Gp16 protein carrying a C-terminal twin-Strep tag was expressed in *S. aureus*. Cultures were induced with aTc and incubated at 37°C for 6 h with shaking at 200 rpm. Cells were harvested by centrifugation at 4000 × *g* for 15 min at 4°C, frozen in liquid nitrogen, and ground into fine powder. The cell powder was resuspended in lysis buffer (100 mM Tris–HCl, pH 8.0, 150 mM NaCl, 1 mM EDTA, 1 mM Phenylmethylsulfonyl fluoride (PMSF)) and lysed by sonication. The lysate was clarified by centrifugation (12 000 × *g*, 20 min, 4°C) and applied to a Strep-Tactin Sepharose column (Cytiva, USA) pre-equilibrated with wash buffer. After extensive washing, bound proteins were eluted with buffer containing 50 mM biotin. Eluted fractions were concentrated and subjected to Liquid Chromatography-Tandem Mass Spectrometry (LC–MS/MS)-based proteomic analysis using an EASY-nLC 1200 UHPLC system coupled to an Orbitrap Q Exactive HF-X mass spectrometer (Thermo Fisher Scientific, USA) operating in data-dependent acquisition mode. Peptides were separated on a C18 analytical column using a linear gradient of 5%–100% acetonitrile in 0.1% formic acid at a flow rate of 600 nl/min. MS/MS spectra were searched against the *S. aureus* protein database to identify Gp16-interacting proteins.

### His-tag pull-down assay

Gp16 was fused to a His-tagged SUMO and cloned into pET28a, while RecA was tagged with an N-terminal Flag tag and cloned into pET21a. Both plasmids were used to co-transform *E. coli* BL21 (DE3). Protein expression was induced with 1 mM IPTG when the cell culture reached an OD_600_ of 0.4. The culture was further incubated for 16 h at 25°C and then harvested by centrifugation at 4000 × *g* for 10 min. The cell pellets were resuspended in lysis buffer (40 mM Tris, pH 8.0, 300 mM NaCl, 10% glycerin, and 10 mM imidazole) and lysed by sonication, followed by centrifugation for 30 min at 12 000 rpm at 4°C. The supernatant was filtered through a 0.22-μm membrane and incubated with Ni-NTA resin (Beyotime, China). After binding, the resin was washed with wash buffer (40 mM Tris, pH 8.0, 300 mM NaCl, 10% glycerin, and 30 mM imidazole). The His-Sumo-Gp16 and the interacting proteins were eluted with elution buffer (40 mM Tris, pH 8.0, 300 mM NaCl, 10% glycerin, and 250 mM imidazole). Elution fractions were analyzed by sodium dodecyl sulfate–polyacrylamide gel electrophoresis (SDS–PAGE), and stained with Coomassie Brilliant Blue R.

### Western blot

Samples from His-tag pull-down assay were loaded onto a 10% polyacrylamide gel, resolved by SDS–PAGE, and transferred to a polyvinylidene fluoride membrane (Bio-Rad, Hercules, CA, USA). Following transfer, membranes were blocked in 5% nonfat dry milk (Bio-Rad) for 1 h at room temperature and then incubated overnight at 4°C with a Flag antibody (1:2000; Sigma). After washing with PBST buffer for three times for 5 min each time at room temperature, the membrane was incubated with a horseradish peroxidase-labeled goat anti-mouse IgG (1:10 000; Beyotime) as the secondary antibody. Enhanced chemiluminescence reagent (Bio-Rad) was used to generate the signal. Image detection and collection are performed using a ChemiDoc imaging system, and the images are analyzed using ImageJ [32].

### Bacterial two-hybrid assay

Protein interactions were analyzed by the BACTH assay [26]. In brief, the “bait protein” and “prey protein” were fused to the pKT25 and pUT18. pKT25 and pUT18 expressing T18 and T25 fusion proteins were co-transformed into *E. coli* BTH101. The transformants were incubated at 30°C for ~48 h before inspection. To quantify protein interactions, co‐transformants were picked and cultured with shaking at 30°C for 16 h in LB containing 0.5 mM IPTG, and β‐galactosidase activities were determined using the standard Miller assay. Results are representative of at least three independent replicates.

### Protein expression and purification

Gp16 and RecA proteins were expressed in *E. coli* BL21 (DE3). The gene encoding *gp16* was cloned into a pET28a vector with a TEV-cleavable N-terminal His_6_-SUMO tag. The *recA* gene was cloned separately into pET28a with a TEV-cleavable N-terminal His_6_ tag. Each plasmid was transformed into *E. coli* BL21. A single colony was amplified in 10 ml LB medium containing kanamycin (50 μg ml^−1^) for 12 h at 37°C with shaking. Cultures were diluted 1:100 into LB medium (50 µg ml^−1^ kanamycin) and cultured at 200 rpm at 37°C until OD_600_ reached ~0.5. Protein expression was induced with 0.3 mM IPTG and grown at 20°C, 200 rpm for 16 h. The culture was centrifuged, and the pellet was resuspended in lysis buffer (20 mM Tris–HCl, pH 8.0, 300 mM NaCl, and 10 mM imidazole). The cells were homogenized and centrifuged at 10 000 × *g* for 20 min at 4°C. The precipitate was incubated with a His-Tag purification resin column (GE Healthcare, USA), followed by protein elution with elution buffer (20 mM Tris–HCl, pH 8.0, 300 mM NaCl, 300 mM imidazole). The His_6_-SUMO tag was cleaved by Tev and removed by passing the samples over Ni-NTA resin (Beyotime, China). The purified protein was dialyzed and the buffer was finally replaced with PBS. Samples were assessed by SDS–PAGE. Protein concentrations were determined by Nanodrop 2000.

### Electrophoretic mobility shift assay

Electrophoretic mobility shift assay (EMSA) was performed using 200 ng of M13mp18 circular single-stranded DNA (ssDNA) or linear double-stranded DNA as substrates. Reactions (20 μl) were carried out in a buffer [25 mM Tris–HCl, pH 7.6, 3 mM potassium glutamate, 10 mM magnesium acetate, and 5% (w/v) glycerin] with increasing concentrations of purified Gp16 protein (0–500 nM). After incubation at 37°C for 40 min, 2 μl of 10× DNA loading buffer was added to each reaction. Samples were resolved on 0.8% agarose gels in 1× TAE buffer at 150 V for 30 min and visualized by nucleic acid staining.

### DNA cleavage assay

DNA substrates included gDNA from *S. aureus* RN4220, gDNA from phage ΦNM1, single-stranded and circular double-stranded DNA from phage M13, and plasmid DNA (pET28a and pUC19). DNA substrates were prepared using extraction kits according to the manufacturers’ instructions, including a bacterial gDNA extraction kit (Tiangen, China), a phage gDNA extraction kit (Zoman Biotechnology, China), and a plasmid extraction kit (Vazyme, China). As described [34], DNA cleavage experiments were carried out in 15 μl reaction volumes containing 200 ng DNA substrate and Gp16 or its mutant protein Gp16^C181A^ (0, 37.5, 75, 150, 300, 600, 1200, or 2400 nM) in a reaction buffer [25 mM Tris–HCl, pH 8.0, 5 mM MgCl_2_, 1 mM DTT, 0.5 mM ATP, and 5% (w/v) glycerin]. Reactions were carried out at 37°C for 0, 10, 30, 60, or 120 min, and then terminated by adding 100 mM EDTA and 2 μl of 10× DNA loading buffer. Reaction products were analyzed by electrophoresis on 0.8% agarose gels in 1× TAE buffer at 150 V for 30 min and visualized by nucleic acid staining. The signal intensity of the initial DNA substrate was quantified using ImageJ software [32]. To calculate the proportion of degraded DNA, the band intensity of the intact substrate in each sample lane was normalized to that of the intact DNA band in the protein-free control. Quantification results are presented as bar graphs showing the mean values from three independent experiments, with error bars indicating the standard error of the mean.

### Determination of oriC/ter ratio in *S. aureus*

The oriC/ter ratio was determined as previously described [35]. *Staphylococcus aureus* strains harboring an empty vector (Vec), a Gp16 expression plasmid, or the Gp16 mutant C181A plasmid were grown overnight in TSB medium supplemented with the appropriate antibiotics at 37°C and diluted 1:100 into fresh medium to OD_600_ of 0.4–0.6. Protein expression was induced with aTc (10 ng ml⁻¹) for 1–2 h. As a positive control, empty vector (Vec) cells were exposed to 0.5 µg ml⁻¹ trimethoprim at OD_600_ of 0.5 to inhibit DNA synthesis. Stationary-phase cultures (OD_600_ = 0.8) were used as reference control. Cells at OD_600_ of 1 were harvested and gDNA was extracted using a Genomic DNA extract kit (Tiangen, China).

Quantitative PCR (qPCR) was performed using 15 ng gDNA in 20 µl reactions containing 0.6 pmol of each primer and 10 µl of 2× SYBR Green Supermix (Bio-Rad) with the following cycling conditions: 95°C for 3 min; 40 cycles of 95°C for 30 s, 60°C for 30 s, and 72°C for 30 s; followed by melt-curve analysis. Primer efficiencies were determined from 5-point, 10-fold serial dilutions of gDNA as E = 10^−1/slope^. oriC/ter ratios were calculated using efficiency-corrected ΔΔCt relative to stationary phase controls:


\begin{eqnarray*}
\frac{\mathrm{ oriC}}{\mathrm{ ter}} = \frac{{E_o}^{({\mathrm{ CT}_c,o}-{\mathrm{ CT}_s,o})}}{{E_t}^{({\mathrm{ CT}_c,t}-{\mathrm{ CT}_s,t})}},
\end{eqnarray*}

where *E_o_* and *E_t_* are the efficiencies of the oriC- and ter-proximal primer pairs, and CT values are from control (C) and sample (S). Values represent the mean of three biological replicates with technical triplicates.

### Quantitative reverse transcription PCR

Total RNA of *S. aureus* cells was extracted using Trizol (Invitrogen, USA) following the manufacturer’s protocol. RNA concentration was quantified by Nanodrop 2000 (Thermo Fisher Scientific, USA) and agarose gel electrophoresis. gDNA in the RNA sample was removed with Genomic DNA Eraser (Promega, USA). For complementary DNA (cDNA) synthesis, 1 μg of total RNA was reverse-transcribed with random primers using the PrimeScript RT Enzyme Reagent Kit (Promega, USA) in a 20-μl reaction. No-RT controls were included to verify the absence of gDNA contamination. Synthesized cDNA samples were diluted five times prior to RT-qPCR. RT-qPCR was accomplished using the SYBR Green (Bio-Rad) following the program: 3 min at 95°C, followed by 40 cycles of 30 s at 95°C, 30 s at 55°C, and 15 s at 72°C. Each 20 μl reaction contained 10 μl SYBR Green mix, 0.4 μM of each primer, and 2 μl of diluted cDNA. Melting curve analysis was conducted to confirm primer specificity. The primers are listed in Supplementary Table S2. Relative transcript levels were calculated using the CT(2^−△△CT^) method, and the 16S RNA was monitored to allow for sample normalization.

### Phage copy number quantification

Quantification of phage copy numbers following infection was performed as previously described [36]. *Staphylococcus aureus* cultures were infected with either ΦNM1 or ΦNM1^Δgp16^ at an MOI of 5. At 0, 10, 30, and 60 min post-infection, cells at OD_600_ of 1 were collected from each time point by centrifugation at 4000 × *g* for 10 min at 4°C. gDNA was extracted from the resulting pellets using the TIANamp Bacteria DNA Kit (Tiangen, China) according to the manufacturer’s instructions. qPCR was performed on a CFX96 Real-Time PCR Detection System (Bio-Rad) using 10 ng of total gDNA per reaction with SYBR Green Master Mix (Bio-Rad) and primer pairs specific for the phage terminase large subunit gene (*gp38*) and the *S. aureus* housekeeping gene *tuf* [37]. Reaction efficiencies for all primer pairs ranged from 95% to 105%, as determined by standard curve analysis. The *tuf* gene served as an internal reference for normalization. Relative phage genome copy numbers were determined by calculating the *gp38*/*tuf* ratio in each sample and normalizing it to the 0 min time point, which was defined as 1. This analysis allowed the determination of fold changes in phage genome copy numbers throughout infection.

### Statistical analysis

GraphPad Prism 8 software was used for statistical analysis of the data. Student’s *t*-test and one-way ANOVA were used to analyze the differences between two or more groups. A *P-*value <.05 was considered statistically significant.

## Results

### Gp16 inhibits *S. aureus* cell growth and promotes phage infectivity

**Figure 1.**
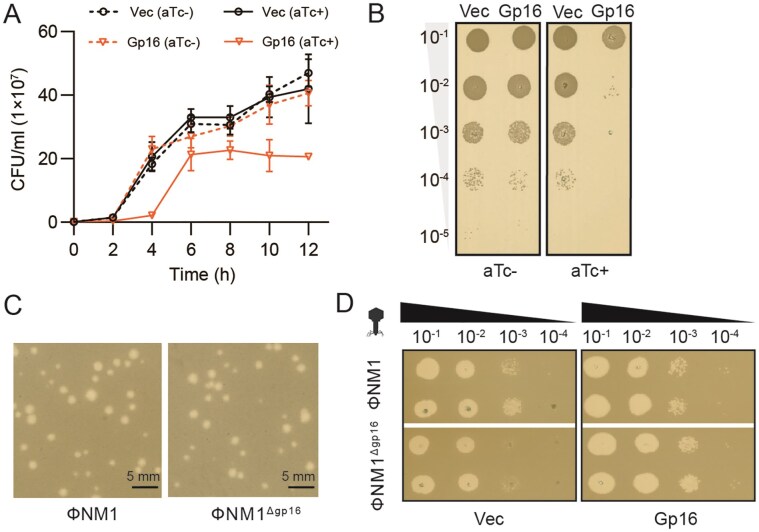
Effects of Gp16 on host growth and ΦNM1 infectivity. (**A**) Effect of Gp16 overexpression on *S. aureus* cell growth compared to empty vector control (Vec). Ten nanograms per milliliter aTc was added to induce the expression of Gp16, and CFU/ml was measured every 2 h for 12 h. (**B**) Comparison of the Gp16-overexpressing strain with the vector control by spotting 10-fold serial dilutions on a TSA plate with ±aTc. (**C**) Plaque morphology of wild-type ΦNM1 and ΦNM1^Δgp16^ on double-layer agar plates. Plaques were generated using the same phage dilutions. (**D**) Ten-fold serial dilution plaque assays were performed to compare the plating efficiency of ΦNM1 and ΦNM1^Δgp16^ on *S. aureus* cells transformed with an inducible plasmid, either empty or encoding *gp16*. The experiments were repeated at least three times.

Previously, we performed a comprehensive screen for early-gene products of ΦNM1 (GenBank accession DQ530359.1) [38] that inhibit cell growth in *S. aureus*. A small membrane protein named Gp11 was characterized to block *S. aureus* cell division by inhibiting peptidoglycan biosynthesis [27]. In addition, another protein named Gp16 (Protein ID ABF73046.1) was found to inhibit cell growth. As shown in Fig. 1A, compared with the empty vector control, overexpression of Gp16 resulted in growth arrest in *S. aureus*. By plating the cultures on TSA plates, inducible overexpression of *gp16* inhibited colony formation (Fig. 1B).

To investigate the role of *gp16*, we generated a *gp16* deletion mutant of phage ФNM1 (ФNM1^Δgp16^). ФNM1^Δgp16^ formed plaques in sizes similar to those of the wild-type phage (Fig. 1C), but its infectivity on double-layer agar plates was reduced with an ~10-fold decrease, as reflected by a significant decrease in EOP (Fig. 1D). This apparent discrepancy likely reflects a reduced probability of productive infection in the absence of *gp16*, whereas infections that successfully proceed through the lytic cycle can still form plaques with morphology comparable to the wild type; and the defect could be complemented in *trans*, since infection of *S. aureus* strains carrying a plasmid-borne *gp16* restored the EOP to wild-type levels (Fig. 1D). These findings indicate that although *gp16* is dispensable for plaque formation, it is required for efficient infection of *S. aureus*.

### Gp16 expressed immediately after phage infection and is important to phage fitness

**Figure 2.**
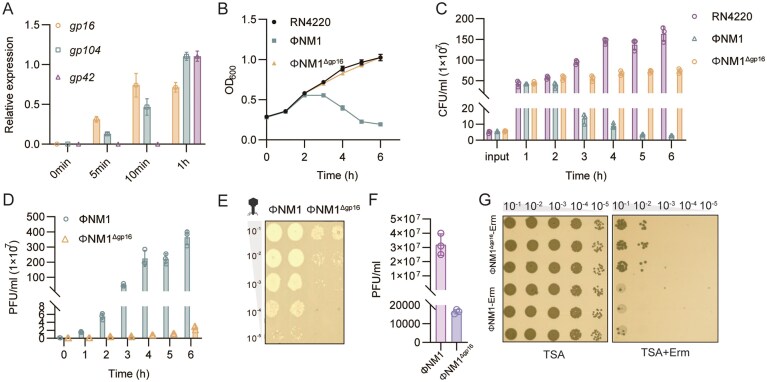
Gp16 is expressed early during phage infection and required for phage fitness. (**A**) Temporal expression profile of *gp16. S. aureus* cells were infected with phage ΦNM1, and transcript levels of *gp16*, the early gene *gp104*, and the late gene *gp42* were measured at 5, 10, and 60 min post-infection using qRT-PCR. Data shown are the average from *n* = 3 experiments. Error bars indicate SEM. (**B**) Growth curves of *S. aureus* cultures infected with wild-type ΦNM1 or ΦNM1^Δgp16^ at an MOI of 0.01. Optical density at 600 nm (OD_600_) was monitored over time. Data shown are the average from *n* = 3 experiments. Error bars indicate SEM. (**C**) Numbers of live cells infected with ΦNM1 or ΦNM1^Δgp16^ at MOI = 0.01, and CFUs were enumerated at indicated time points. Data shown are the average from *n* = 3 experiments. Error bars indicate SEM. (**D**) Phage production was determined from culture supernatants of *S. aureus* infected with wild-type ΦNM1 or ΦNM1^Δgp16^ at MOI = 0.01. Data shown are the average from *n* = 3 experiments. Error bars indicate SEM. (**E**) Phage production after mitomycin C induction. Lysogens of wild-type ΦNM1 or ΦNM1^Δgp16^ were induced with mitomycin C, and progeny phages were quantified by plaque assay at the indicated times. The experiments were repeated at least three times. (**F**) Numbers of phage release from lysogens show in panel (E). (**G**) Lysogen formation assay. An erythromycin resistance marker (*ermC*) was integrated into wild-type ΦNM1 and ΦNM1^Δgp16^ genomes, and the number of lysogens was quantified in *S. aureus* following infection at MOI = 10. The experiments were repeated at least three times.

Since *gp16* deletion affected infection efficiency, we next asked at which stage *gp16* is expressed and how it contributes to phage propagation. *gp16* transcripts were detected 5 min post-infection at levels comparable to the early gene *gp104* [39], confirming that *gp16* is an early gene, whereas *gp42* [40], which is required for phage particle assembly and is expressed late in the infection cycle, was transcribed after 1 h of infection (Fig. 2A).

To assess the functional consequences of *gp16* deletion, we compared host survival and phage propagation in liquid culture. Growth curve analyses across a range of MOIs revealed that wild-type ФNM1 caused a rapid and substantial decline in optical density, whereas ФNM1^Δgp16^ infection allowed a much larger fraction of the population to persist (Supplementary Fig. S1). At an MOI of 0.01, the OD_600_ profile of ФNM1^Δgp16^-infected cultures was nearly indistinguishable from that of uninfected controls. More detailed measurements at this MOI showed that, beginning at 3 h post-infection, cultures infected with wild-type ФNM1 exhibited a pronounced loss of cell density relative to ФNM1^Δgp16^ (Fig. 2B). Viable cell counts were reduced ~25-fold in the wild-type infection compared with the mutant at 6 h (Fig. 2C). In line with this, despite the higher host survival, ФNM1^Δgp16^ produced nearly 100-fold fewer progeny phages than wild-type ФNM1 in 6 h after infection (Fig. 2D), highlighting a critical role of *gp16* in virion production.

**Figure 3.**
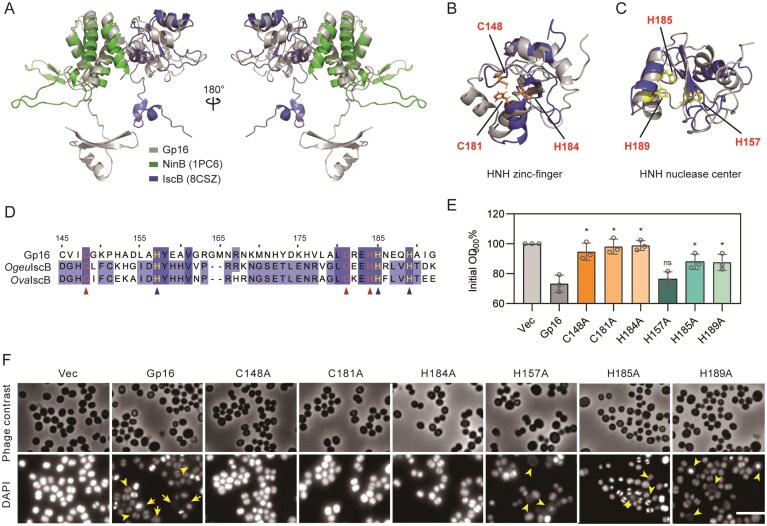
Gp16 acts as a nuclease to induce DNA condensation in *S. aureus*. (**A**) AlphaFold-predicted structure of Gp16 compared with the protein NinB (PDB: 1PC6) and IscB (PDB: 8CSZ). Crystal structure of Gp16 is shown by 180° rotation around the *y* axis. Gp16, gray; NinB, green; OgeuIscB, blue. (**B**) Cartoon representation of the Zinc finger in the HNH domain of Gp16 compared with IscB, the active sites C148, C181, and H184 are highlighted in red. (**C**) Cartoon representation of the nuclease center in HNH domain of Gp16 compared with IscB, the HNH nuclease active sites H157, H185, and H189 are highlighted in red. (**D**) Amino acid sequence alignment of HNH active sites. Red triangles mark the HNH zinc-finger active sites, and blue triangles mark the HNH nuclease center active sites. Sequence is numbered according to Gp16 amino acid sequence. (**E**) The growth of *S. aureus* cells expressing Gp16 or its zinc-finger active site mutants, nuclease center active site mutants relative to the control strain containing empty vector after 4 h cultivation with 10 ng ml^−1^ aTc. The *P-*values were calculated by Student’s *t*-test, ∗*P* < .05, and ns (*P* > .05). Data shown are the average from *n* = 3 experiments. Error bars indicate SEM. (**F**) *Staphylococcus aureus* cells expressing Gp16 or its point mutants were observed under phase contrast and fluorescence microscopy of cells stained with DAPI. The arrows indicate the cells with weak or condensed nucleoid, enlarged cells, or cells lacking detectable nucleoids. Scale bars, 2.5 µm. The experiments were repeated at least three times.

In addition, treatment of lysogens with MMC to induce prophage resulted in significantly different phage yields between wild-type and mutant strains. Following MMC treatment, infective phages were detected 0.5 h after induction and reached ~3.2 × 10^7^ PFU/ml for wild-type ФNM1, whereas ФNM1^Δgp16^ yielded only ~1.65 × 10^4^ PFU/ml, demonstrating that although viable, the mutant was severely impaired in virion release (Figs 2E and 3F). To enable quantification of lysogens carrying a stably integrated prophage, an erythromycin resistance gene (*ermC*) was introduced into ΦNM1 and ΦNM1^Δgp16^ (Supplementary Fig. S2A). The effect of *ermC* insertion on phage infectivity was assessed. Infections of *S. aureus* at MOIs of 0.01 and 10 yielded trends consistent with those observed for phages lacking *ermC* (Supplementary Figs S1 and  S2), demonstrating that the insertion did not impair phage infectivity. Notably, cultures infected with ΦNM1^Δgp16^-Erm produced a greater number of lysogens compared with ΦNM1 at MOI = 10 (Fig. 2G). Since loss of *gp16* diminishes phage replication efficiency, the infection outcome becomes biased toward lysogeny, explaining the increased formation of lysogens observed here. Taken together, these results indicate that, although *gp16* is dispensable for plaque formation, it contributes to efficient phage induction, virion release, and robust inhibition of host growth, thereby enhancing overall phage fitness.

### Gp16 is a member of the HNH-endonuclease family

To further explore the function of Gp16, we next performed a PSI-BLAST search using the *gp16* sequence as a query. Our results revealed that Gp16 contains a CXXC motif (Cys145-Val146-Ile147-Cys148) and a CXXH motif (Cys181-Arg182-Glu183-His184), which is found in HNH endonucleases of subclass 6. However, this analysis did not reveal any such characterized proteins. To investigate the conformational dynamics of Gp16, we used AlphaFold 3 to predict its potential alternate structures [31, 41]. The predicted structure of Gp16 consists of two distinct domains: the N-terminal domain shows a strong structural resemblance to the homologous recombination mediator NinB (PDB: 1PC6) [42], while the C-terminal domain resembles the HNH nuclease IscB (PDB: 8CSZ) [43] (Fig. 3A). Notably, the C-terminal nuclease domain contains conserved HNH zinc-finger and nuclease center motifs revealed by structural alignment (Fig. 3B and C).

The predicted structural model suggested that Gp16 contains a conserved HNH nuclease domain, prompting us to examine its functional contribution. To this end, we generated single amino acid substitutions in residues predicted to coordinate zinc ions (C148A, C181A, and H184A) within the HNH-type zinc-finger motif and in catalytic residues (H157A, H185A, and H189A) within the nuclease center, which are highly conserved among IscB-like endonucleases (Fig. 3D). Expression of wild-type Gp16 inhibited *S. aureus* cell growth, whereas mutations in the zinc-finger motif (C148A, C181A, and H184A) completely abolished the growth inhibition (Fig. 3E). The H157A substitution in the HNH nuclease motif did not affect the growth inhibition, while substitutions at the nuclease center (H185A and H189A) only partially relieved growth inhibition, suggesting that the catalytic residues are important but not solely responsible for the inhibitory phenotype (Fig. 3E).

Given its putative nuclease structure, we next examined whether Gp16 expression affects nucleoid organization within the cell. Overexpression of wild-type Gp16 caused a subpopulation of cells to enlarge, as visualized by phase-contrast microscopy. DAPI staining revealed pronounced DNA condensation within these enlarged cells, consistent with previous observations [35], even in cells with no nucleus (Fig. 3F). In contrast, the C148A, C181A, and H184A mutants did not induce DNA condensation and displayed cell morphologies comparable to the control, consistent with their inability to inhibit bacterial growth (Fig. 3F). Substitutions at the HNH nuclease center (H185A and H189A) partially alleviated growth inhibition but still induced DNA condensation, whereas the H157A mutation did not affect bacterial growth yet retained the DNA-condensed phenotype, indicating that disruption of these catalytic residues weakens but does not abolish the nuclease-mediated effects (Fig. 3F). Collectively, these results demonstrate that the catalytic integrity of the HNH nuclease domain is essential for Gp16-induced inhibition of *S. aureus* growth and perturbation of nucleoid organization.

### Gp16 functions as a nickase *in vitro*

**Figure 4.**
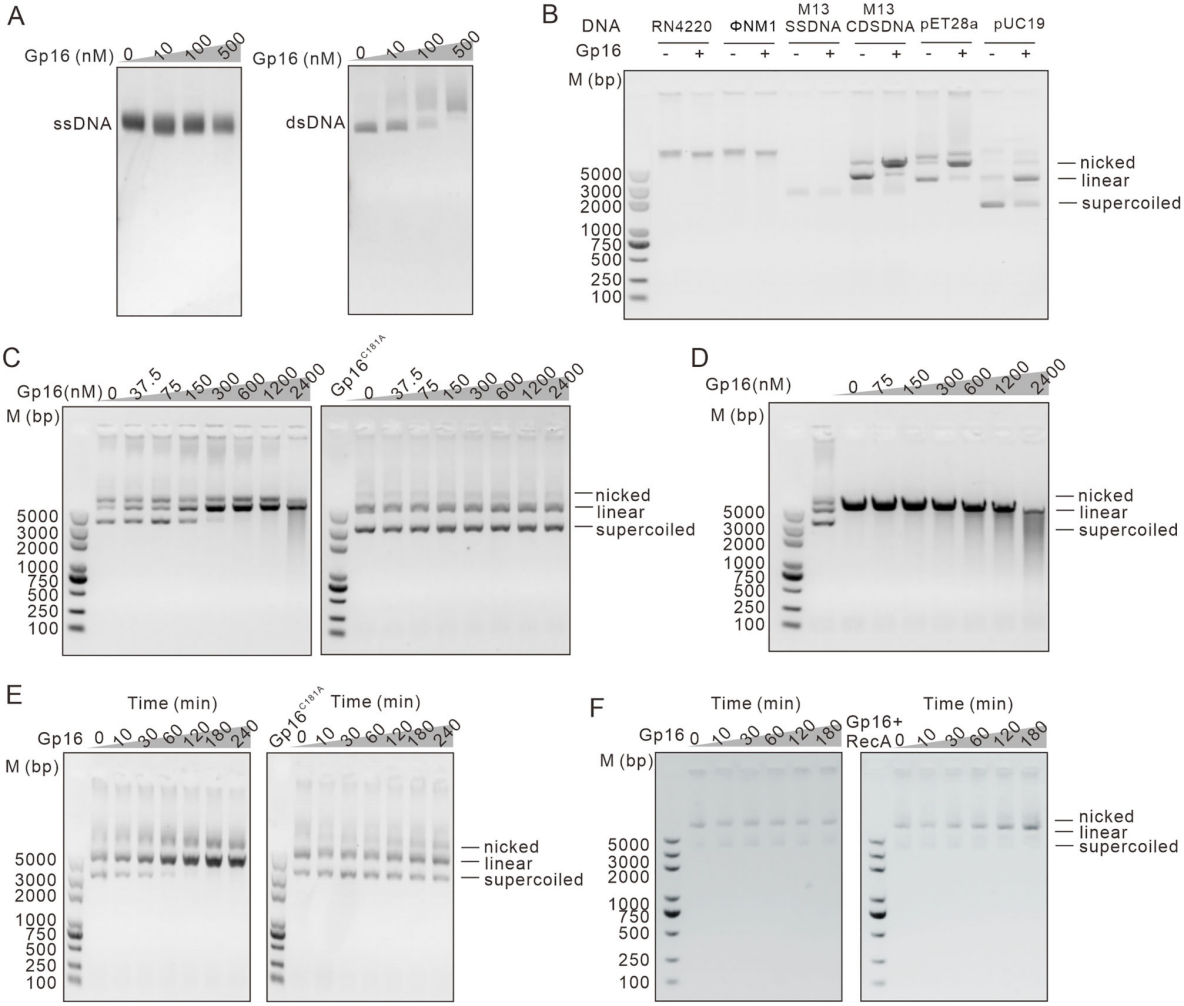
Time- and dose-dependent nuclease activity of Gp16. (**A**) EMSAs were run on a 0.8% agarose gel for the ssDNA or dsDNA. The concentrations of Gp16 added were shown in the figure. (**B**) Gp16 displays a plasmid-biased nuclease activity. Nuclease activities of purified Gp16 toward DNA substrates of different origins and forms are performed. Reaction mixtures contained gDNA from *S. aureus* RN4220, gDNA from phage ΦNM1, circular ssDNA and dsDNA from phage M13, and plasmids pET28a and pUC19. (**C**) Cleavage activity of Gp16 and its mutant Gp16^C181A^ on the plasmid substrate pET-28a at concentrations ranging up to 2400 nM. (**D**) Cleavage activity of Gp16 and on the linear DNA substrate pET-28a at concentrations ranging up to 2400 nM. (**E**) Time points from a nuclease assay of Gp16 and its mutant Gp16^C181A^ on the plasmid substrate pET-28a. Gp16 or Gp16^C181A^ concentration was constant at 150 nM. (**F**) RecA enhances the nuclease activity of Gp16. pET-28a was incubated with 75 nM Gp16 in the presence or absence of 37.5 nM RecA, and DNA cleavage was monitored over 3 h. All the reaction progression is shown by the transition of the bottom band (supercoiled DNA) to the top (nicked) and middle (linear) bands. All reactions were carried out at 37°C for the indicated times, and reaction products were analyzed by agarose gel electrophoresis. The experiments were repeated at least three times.

Structural prediction analysis indicated that the C-terminal HNH domain of Gp16, with conserved zinc-finger motifs, may facilitate nucleic acid binding. To test this, we performed EMSA using M13mp18 ssDNA and dsDNA substrates at 0–500 nM Gp16. No binding was observed with ssDNA, whereas dsDNA exhibited clear gel shifts with increasing Gp16 concentrations (Fig. 4A), demonstrating selective dsDNA binding. Having demonstrated that the catalytic integrity of the HNH domain is essential for Gp16-mediated inhibition of *S. aureus* cell growth and DNA condensation *in vivo*, we therefore sought to directly assess its nuclease activity *in vitro*. Gp16 was tested against various DNA substrates and showed a strong preference for plasmid DNA, indicating plasmid-biased nuclease activity (Fig. 4B).

To further characterize this activity, the plasmid substrate pET28a was incubated with increasing concentrations of purified Gp16. At 150 nM, the supercoiled plasmid was largely converted into its linear form, while higher concentrations resulted in progressive accumulation of nicked and degraded DNA species, with extensive degradation observed at 2.4 μM (Fig. 4C). Quantification showed that ~70% of the plasmid DNA was cleaved at 300 nM Gp16 (Supplementary Fig. S3A). In contrast, the catalytic mutant Gp16^C181A^ exhibited no detectable nuclease activity toward plasmid DNA under identical conditions (Fig. 4C), and its cleavage efficiency did not vary with protein concentration (Supplementary Fig. S3A). When linearized plasmid DNA was used as the substrate, 300 nM Gp16 induced only slight degradation after 1 h, whereas higher concentrations caused extensive digestion (Fig. 4D), with ~60% of the DNA degraded at 2.4 μM (Supplementary Fig. S3B). Time-course assays further revealed that Gp16 rapidly cleaved plasmid DNA in a time-dependent manner, converting supercoiled DNA to its linear form within 10 min and causing progressive degradation with prolonged incubation (Fig. 4E). After 2-h incubation with 150 nM Gp16, ~95% of the supercoiled plasmid was cleaved, whereas Gp16^C181A^ remained inactive throughout (Supplementary Fig. S3C). Collectively, these results demonstrate that Gp16 functions as a nickase whose activity depends on both protein concentration and reaction time, and that its catalytic cysteine residue (C181) is essential for DNA cleavage.

### Host protein RecA is required for Gp16 mediated growth arrest

**Figure. 5.**
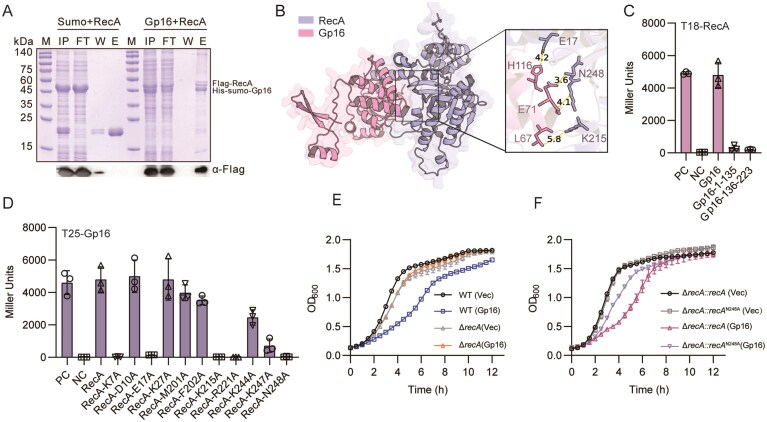
Gp16 interacts with RecA and mediates growth defect. (**A**) His pull-down assay of Gp16 and RecA. SDS–PAGE gel analysis of co-purifications of Gp16, Gp16, and RecA stained with Coomassie blue. Antibody against the Flag tag was used for western blot. (**B**) Structural model of the Gp16–RecA complex predicted by AlphaFold3, with the model confidence score for protein–protein interaction is 0.81. Potential interactions involving E17, K215, and N248 are highlighted. (**C**) β-Galactosidase activity in the BACTH assay for truncated Gp16 and RecA interaction. Data shown are the average from *n* = 3 experiments. Error bars indicate SEM. (**D**) Validation of key RecA residues involved in Gp16 binding. Site-directed mutations of RecA residues were performed, and interaction was assessed by BATCH assay. Data shown are the average from *n* = 3 experiments. Error bars indicate SEM. (**E**) Gp16-mediated growth arrest depends on RecA. The growth curves for cells expressing Gp16 in either wild type (WT) or Δ*recA* with 10 ng ml^−1^ aTc induction. The bacteria with empty vector as control (Vec). OD_600_ of *n* = 3 biological replicates are shown. (**F**) Growth curves of bacteria expressing Gp16 or empty vector in Δ*recA::recA* or Δ*recA::recA*^N248A^ complemented strains. OD_600_ of *n* = 3 biological replicates are shown.

To identify the cellular target of Gp16, an *in vivo* pull-down assay was performed. Proteins co-purified with Gp16 were analyzed by liquid chromatography–tandem mass spectrometry (LC–MS), which revealed RecA as a potential Gp16-interacting partner. To confirm this interaction, an *in vitro* His pull-down assay was conducted. Following Ni-NTA affinity chromatography, Flag-RecA was detected in the elution fraction only in the presence of His-SUMO-Gp16, confirming a specific interaction between Gp16 and RecA (Fig. 5A).

To gain further insight into the molecular basis of this interaction, AlphaFold3 was employed to predict the interaction of Gp16–RecA complex (Supplementary Fig. S4). The structural model generated by AlphaFold3 revealed an extensive interaction surface between the two proteins with a high confidence score of 0.81, supporting the reliability of the predicted complex (Fig. 5B). Consistent with these results, a BATCH assay further verified the interaction of Gp16–RecA, whereas neither the N-terminal nor the C-terminal truncations of Gp16 retained binding activity, suggesting that both termini are required for complex formation (Fig. 5C). Guided by the structural model, point mutations were introduced into several residues of RecA to assess their contribution to Gp16 binding. As shown in Fig. 5D, substitutions at K7, E17, K215, R224, K247, and N248 markedly reduced interaction with Gp16, identifying these residues as critical determinants of binding.

Since RecA is nonessential for bacterial growth, Gp16 must inhibit growth by co-opting, rather than merely inactivating, RecA. We therefore tested whether Gp16 requires a functional RecA protein for its activity. Indeed, Gp16 expression failed to inhibit the growth of a Δ*recA* mutant, but inhibition was restored upon complementation with wild-type *recA* (Fig. 5E and F). The RecA ^N248A^ mutant was introduced as this residue lies closest to Gp16 in the predicted interface (distance ≈ 3.6 Å) (Fig. 5B). Crucially, this growth inhibition was abolished in a strain expressing a RecA variant (RecA^N248A^) that is defective in Gp16 binding (Fig. 5F), demonstrating that the physical interaction between Gp16 and RecA is essential. This RecA-dependent mechanism is further supported by *in vitro* findings that RecA enhances the DNA cleavage activity of Gp16 (Fig. 4F), suggesting that the RecA interaction not only mediates cellular growth arrest but also potentiates Gp16’s enzymatic function.

### Gp16 promotes phage infection via RecA and suppresses host replication

**Figure 6.**
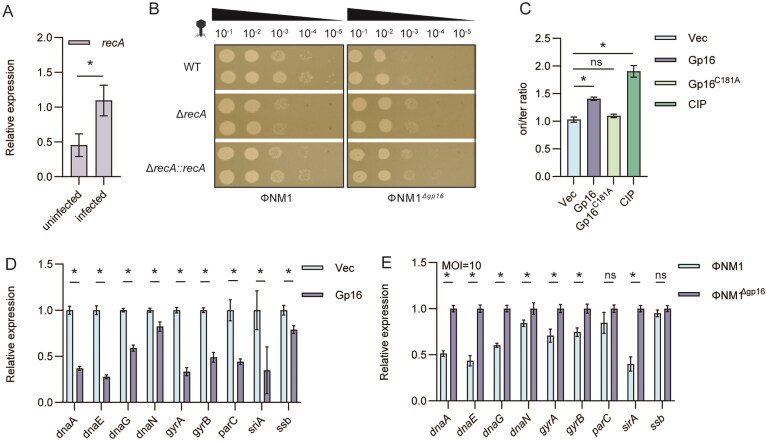
Gp16 hijacks RecA to inhibit host replication. (**A**) qRT-PCR analysis of *recA* expression in *S. aureus* cells collected 10 min after ΦNM1 infection (MOI = 10). Data from *n* = 3 experiments are shown. Error bars indicate SEM. (**B**) Ten-fold serial dilutions of phage ΦNM1 and ΦNM1^Δgp16^ were plated onto lawns of wild type, Δ*recA*, and *recA*-complemented strain Δ*recA::recA*. (**C**) oriC/ter ratio determined by real-time PCR in cells expressing *gp16* or treated with ciprofloxacin. Data shown are the average from *n* = 3 experiments. Error bars indicate SEM. (**D**) qPCR analysis of replication-associated genes (*dnaA, dnaE, dnaN, gyrA, gyrB, parC, sirA*, and *ssb*) in cells overexpressing *gp16*. Data shown are the average from *n* = 3 experiments. Error bars indicate SEM. (**E**) qPCR analysis of replication-associated genes in ΦNM1 and ΦNM1^Δgp16^ strains 10 min post ΦNM1 infection (MOI = 10). Data shown are the average from *n* = 3 experiments. Error bars indicate SEM.

To investigate the role of RecA during phage infection, we first measured *recA* expression at 10 min post-infection, a time point corresponding to the onset of robust *gp16* transcription. The phage infection significantly upregulated *recA* compared to uninfected cells (Fig. 6A). The importance of RecA was further supported by the EOP assay: deletion of *recA* markedly resulted in an ~10-fold reduction in EOP during ΦNM1 infection, whereas complementation restored it to wild-type levels (Fig. 6B). In line with these observations, knocking out *gp16* significantly reduced EOP; however, this reduction was reversed when RecA was absent, indicating that Gp16 depends on RecA to function (Fig. 6B). This reversal supports a model in which Gp16 acts through RecA during early infection to modulate host processes in favor of phage replication.

Cell elongation and DNA condensation are hallmarks of replication arrest [44]. To test whether Gp16 disrupts DNA replication, we measured the oriC/ter ratio of the *S. aureus* chromosome by real-time PCR. As a positive control, cells treated with the DNA synthesis inhibitor ciprofloxacin [45], which showed an ~two-fold increase in the oriC/ter ratio (Fig. 6C). Likewise, Gp16 expression resulted in a significant increase in the oriC/ter ratio compared with the vector control, whereas the C181A mutant showed no effect (Fig. 6C), indicating that Gp16 blocks replication fork progression and prevents completion of chromosomal replication. qPCR analysis further confirmed that *gp16* overexpression suppresses the transcription of key replication-associated genes, including *dnaA, dnaE, dnaN, gyrA, gyrB, parC*, and *sirA* (Fig. 6D). To examine the physiological role of Gp16 during phage infection, we sampled cells 10 min after infection at an MOI of 10, when Gp16 is rapidly expressed. Notably, deletion of *gp16* resulted in a significant increase in the transcription of replication-associated genes, consistent with the inhibitory effect observed upon *gp16* overexpression (Fig. 6E). Together, these findings demonstrate that *gp16* is expressed immediately upon infection, where it suppresses host DNA replication and promotes host DNA condensation.

To determine whether Gp16-mediated suppression of host replication benefits phage propagation, we monitored phage replication. Deletion of *gp16* reduced phage replication by ~1.9-fold at 30 min and 5.8-fold at 1 h post-infection (Supplementary Fig. S5). Consequently, Gp16-mediated shutdown of host replication and recruitment of RecA not only inhibit host growth but also enhance phage replication. This dual activity underscores how Gp16 simultaneously suppresses the host and promotes phage infection.

## Discussion

The evolutionary arms race between bacteria and their phages is driven by early phage genes that facilitate host takeover and host defenses that counteract this process [46, 47]. Although most early phage genes are nonessential, they are thought to fine-tune infection or function under specific conditions, yet the majority of these remain functionally uncharacterized [48]. In this study, we identify Gp16 as an early gene product that enhances phage fitness while inducing growth arrest in *S. aureus*. Unlike previously described phage proteins that directly target essential bacterial factors [27], Gp16 employs an indirect strategy by co-opting the nonessential host protein RecA to favor phage replication. Our findings reveal that Gp16 promotes phage propagation through RecA while simultaneously suppressing host DNA replication, thereby providing a mechanistic framework for phage exploitation of host functions and offering new insights into the evolutionary dynamics of phage–host interactions.

Building upon these findings, Gp16 illustrates the functional role of phage-encoded nucleases in manipulating host DNA metabolism to optimize infection outcomes. Our characterization of Gp16 aligns with recent studies highlighting nucleases as pivotal players in the molecular arms race between bacteria and their phages. While bacterial nucleases, such as the Class 1 OLD family nuclease Vc OLD from *V. cholerae*, act as defense effectors to restrict phage replication, an effect counteracted by the ICP1-encoded inhibitor Oad1[49], Gp16 represents a distinct paradigm. Unlike bacterial immunity functions, Gp16, a phage-encoded nuclease, relies on the host RecA protein to promote phage replication, showcasing a cooperative interaction between a phage effector and a host DNA repair factor. This mechanism contrasts sharply with bacterial RM and CBASS-associated nucleases, which aim to protect the host by targeting invading phage genomes [2]. These findings collectively underscore the dual roles that nucleases play in the conflict between bacteria and phages, acting either as host defense components or as phage-encoded effectors that modify host DNA metabolism. This duality broadens the conceptual framework of nuclease-mediated interactions and illustrates how phages exploit host DNA maintenance machinery to coordinate host suppression with efficient phage propagation. Given that ΦNM1 is a temperate phage [38], we explored how Gp16 modulates the infection outcome between lysis and lysogeny. Temperate phages can follow either a lysogenic or the lytic lifestyle [50]. The deletion of *gp16* significantly impairs phage replication and increases the frequency of lysogen formation, suggesting that Gp16 biases the infection outcome toward the lytic cycle. This bias is critical for phage fitness as it ensures efficient progeny production; conversely, the absence of Gp16 supports stable prophage integration. Prophages are known to drive lysogenic conversion, altering host phenotypes such as virulence [51], and contributing to genome diversification and adaptive evolution by disrupting key functions or facilitating horizontal gene transfer [52]. Therefore, changes in lysogeny frequency can profoundly impact host–phage interactions. By adjusting the balance between lysis and lysogeny, Gp16 not only enhances phage reproductive success but may also indirectly shape the long-term evolutionary path of its bacterial host.

To gain mechanistic insight into how Gp16 influences host growth and phage replication, we analyzed its structural features, identifying it as a canonical HNH-like nuclease. Gp16 and its homologs in related Dubowirus phages constitute a conserved early-gene strategy that inhibits *Staphylococcus* growth and biases infection toward the lytic cycle, thereby enhancing phage replication efficiency and progeny production. The widespread conservation of *gp16*-like genes suggests that such host-targeting mechanisms provide a competitive advantage and may contribute to the evolutionary success of the genus. HNH-like proteins exhibit an evolutionarily conserved structural and functional organization, consisting of two modules: an N-terminal α-helical structure and a more conserved C-terminal DNA binding domain and catalytic domain [53]. The predicted structure of the Gp16 C-terminal domain, resembling that of IscB, displays an active site architecture characteristic typical of HNH endonucleases with the canonical ββ-α fold. Site-directed mutagenesis of residues within this active site abolished growth inhibition mediated by Gp16, suggesting that the C-terminal HNH nuclease domain, and particularly its zinc-finger motif, is essential for its nuclease activity. AlphaFold modeling further suggests that the N-terminal region of Gp16 adopts a fold similar to NinB. Given that NinB acts as a recombination mediator that antagonizes RecFOR and facilitates RecA filament formation on SSB-coated ssDNA [54], it is plausible that the N-terminal domain of Gp16 plays a similar role in modulating host recombination processes, potentially coordinating DNA nicking with RecA-dependent pathways, though this hypothesis requires experimental validation.

During phage infection, RecA is activated in response to replication stress and DNA damage, initiating homologous recombination and DNA repair pathways [55]. Our data reveal that *gp16* is transcribed immediately after the onset of infection, ensuring that Gp16 is present alongside RecA activation. Beyond its canonical role in host repair, RecA also facilitates the cleavage of phage-encoded repressors, allowing transcription from early promoters and initiating the lytic cycle [56]. Gp16 appears to alter this early activation event by interacting with RecA, redirecting its activity from host DNA repair to phage genome replication and recombination. Concurrently, Gp16 exploits RecA to suppress host growth, further shifting the intracellular balance toward phage propagation. This dual functionality underscores the critical role of RecA availability in determining the infection outcome, as reduced RecA activity significantly diminishes phage DNA replication and phage yield [57]. Together, these findings highlight how ФNM1 ensures a productive infection by coupling early *gp16* expression with RecA-dependent reprogramming of host cellular pathways. Gp16 interacts with the host RecA and simultaneously functions as a nuclease, embodying a multifunctional strategy to modulate host DNA metabolism. Among RecA-dependent nucleases, the P1 Ref protein serves as a well-characterized example: although it has inherently weak endonuclease activity, its endonuclease activity is greatly enhanced when recruited to ssDNA by RecA filaments, leading to programmable double-strand breaks [21–23]. In contrast, Gp16 possesses intrinsic nuclease activity and is capable of cleaving plasmid DNA *in vitro* independent of RecA. While Ref protein enhances RecA-dependent recombination *in vivo* through an unknown mechanism [58], Gp16 stands out by inhibiting host growth and markedly impairing phage genome replication and progeny production. This reveals a broader physiological role that connects RecA interaction to host inhibition and efficient phage propagation. Functionally, Gp16 resembles the phage protein 015, which introduces DNA nicks that block replication fork progression and suppress host cell growth [15]. Our findings suggest that Gp16 exerts a similar activity, consistent with its role in perturbing bacterial DNA metabolism. However, whether Gp16 cleaves specific targeting oligonucleotides at preferred sites remains to be determined, leaving open questions for future studies.

Phages can also conserve energy for infection by shutting off host processes, such as host replication and cell division [39, 59]. Our data indicate that Gp16 contributes to this strategy by inducing condensation of host DNA, a phenotype likely to impede replication. Although Gp16 displays nuclease activity independently of RecA *in vitro*, its inhibitory effect *in vivo* seems to rely on host factors, underscoring how the phage exploits the cellular environment to maximize DNA degradation. Mechanistically, overexpression of Gp16 increases the oriC/ter ratio, indicating replication fork collapse and subsequent DNA condensation. This effect is similar to the action of antibiotics such as ciprofloxacin and trimethoprim, which disrupt replication by uncoupling initiation from elongation, leading to stalled forks and elevated origin-proximal copy numbers [15, 35].

By rapidly halting host replication, Gp16 may prevent competition for critical resources such as dNTPs and replication enzymes, while simultaneously preempting host repair pathways from counteracting DNA damage. This DNA condensation represents a defensive preemptive strategy by which the phage ensures early control over host chromosome metabolism to favor phage replication. Several phage proteins have been shown to directly target bacterial replication machinery. For example, the N4 gp8 protein inhibits the clamp loader subunit of DNA polymerase II, whereas phage LUZ24 Igy protein targets the DNA gyrase subunit B of *Pseudomonas aeruginosa* [12, 39, 59], but the precise role of these inhibitors during phage replication remains elusive. Instead, Gp16 employs an indirect, RecA-dependent mechanism, highlighting a distinct route through which phages reprogram host replication.

In summary, our findings uncover a distinct mechanism by which a phage-encoded nuclease exploits a host regulatory hub to coordinate bacterial shutdown with phage propagation. By engaging RecA, a central component of bacterial stress signaling, Gp16 couples host replication arrest with phage genome amplification, exemplifying functional integration between phage and host systems. This dual control not only enhances phage fitness but also highlights the evolutionary versatility of phage effectors in rewiring essential bacterial pathways. More broadly, our study provides a conceptual framework for identifying phage-encoded modulators that alter bacterial physiology, offering new insights into microbial interactions and potential targets for antibacterial innovation.

## Supplementary Material

gkag024_Supplemental_Files

## Data Availability

The MS data have been deposited to the ProteomeXchange Consortium via the iProX repository (www.iprox.org) with the dataset identifier PXD069936. The sequences of all modified plasmid vectors have been deposited in Zenodo (DOI: 10.5281/zenodo.17937894). All other data are available in the manuscript or supplementary information.
