# Supplementary material for: Bacteriophage deploys a RecA-dependent nuclease to inhibit Staphylococcus aureus replication and promote phage propagation
Source: Nucleic Acids Res. 2026 Jan 20;54(2):gkag024. doi: 10.1093/nar/gkag024 (PMC12817073; doi:10.1093/nar/gkag024)
Supplement: gkag024_Supplemental_Files [file gkag024_supplemental_files.zip › Supplemental file.pdf]

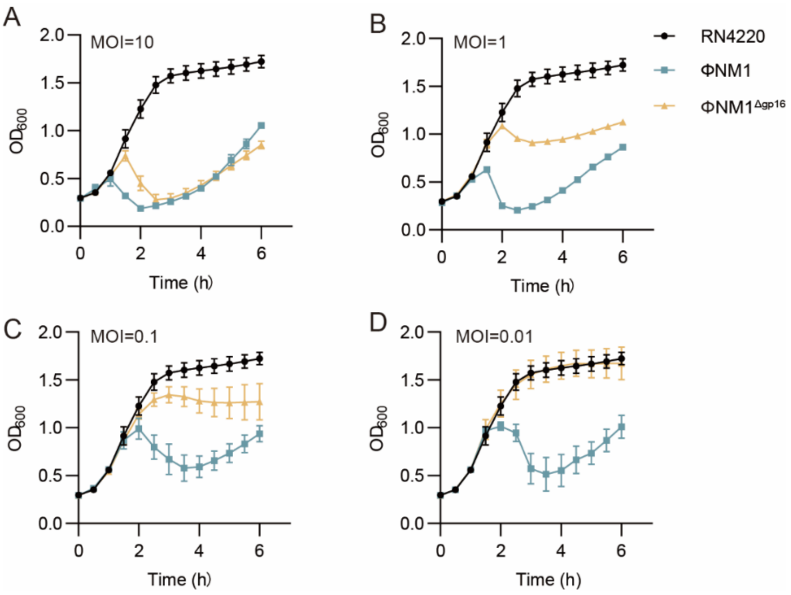

Figure S1. Effect of *gp16* deletion on bacterial growth. (**A**) Growth curves of *S. aureus* cultures infected with wild-type ΦNM1 or ΦNM1$^{\Delta gp16}$ at a multiplicity of infection (MOI) of 10, (**B**) 1, (**C**) 0.1, or (**D**) 0.01. Cell density (OD_{600}) was measured every 30 min at 37 °C using a shaking plate reader. Data represent the mean ± SD from three independent experiments.

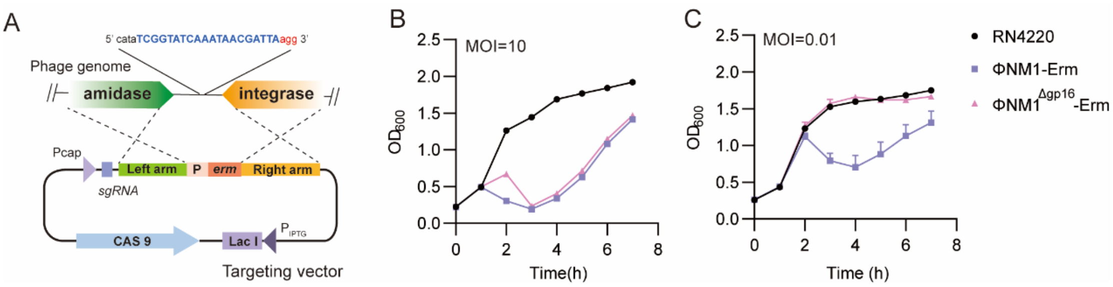

Figure S2. Construction and validation of ΦNM1 and ΦNM1$^{\Delta gp16}$ carrying an erythromycin resistance marker (*ermC*). (**A**) Schematic representation of the insertion of the *ermC* cassette into the ΦNM1 and ΦNM1$^{\Delta gp16}$ genomes to enable quantification of lysogens carrying stably integrated prophages. (**B & C**). Assessment of the effect of *ermC* insertion on phage infectivity. *S. aureus* cultures were infected with ΦNM1-Erm and ΦNM1$^{\Delta gp16}$-Erm at a MOI of 10 (B) or 0.01 (C). Cell density (OD_{600}) was measured every 30 min at 37 °C using a shaking plate reader. Data represent the mean ± SD from three independent experiments.

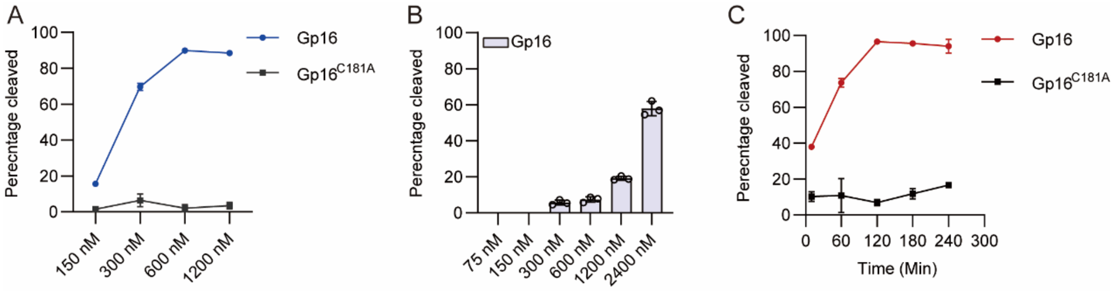

Figure S3. Cleavage efficiency of Gp16 and its mutant Gp16^{C181A}. (**A**) Quantification of plasmid DNA cleavage corresponding to Fig. 4B. The relative intensity of intact DNA bands was measured using ImageJ, and the percentage of degraded DNA was calculated by comparison with the protein-free control. (**B**) Quantification of DNA degradation corresponding to Fig. 4C. When linearized plasmid DNA was used as the substrate. (**C**) Quantification of plasmid DNA degradation corresponding to Fig. 4D. pET28a was incubated with 150 nM Gp16 or its mutant Gp16^{C181A} for the indicated times, and the remaining intact DNA was quantified using ImageJ.

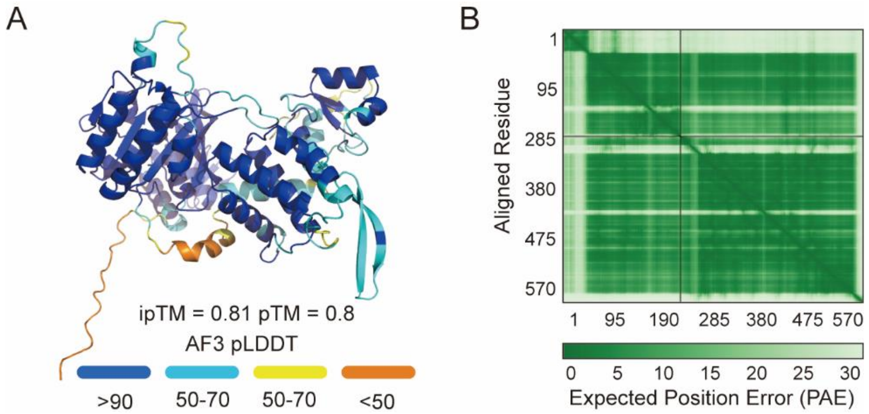

Figure S4. AlphaFold3 prediction of the Gp16-RecA complex. (**A**) AlphaFold3-predicted structure of the Gp16-RecA complex colored by pLDDT confidence scores (dark blue: very high; cyan: high; yellow: low; orange: very low). The ipTM and pTM for protein-protein interaction are depicted below. (**B**) Predicted aligned error (PAE) matrix for the AlphaFold3 predicted interaction between Gp16 and RecA.

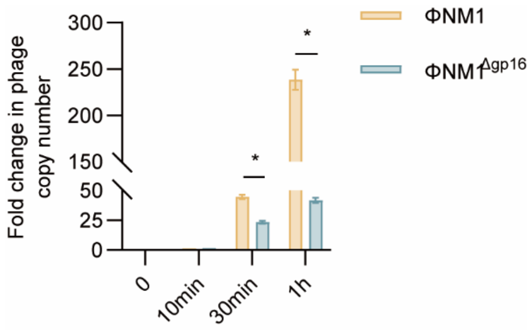

Figure S5. The effect of *gp16* on phage replication. Absolute quantification of phage replication using *gp38* as a phage marker, comparing wild-type and $\Phi NM1^{\Delta gp16}$ phages at 30 min and 1 h post infection.
